# Healthcare-Associated Respiratory Syncytial Virus in Children’s Hospitals

**DOI:** 10.1093/jpids/piad030

**Published:** 2023-05-05

**Authors:** Lisa Saiman, Susan E Coffin, Larry K Kociolek, Danielle M Zerr, Aaron M Milstone, Margaret L Aldrich, Celibell Y Vargas, Giovanny Zapata, Morgan A Zalot, Megan E Reyna, Amanda Adler, Annie Voskertchian, Emily R Egbert, Luis Alba, Sonia Gollerkeri, Madelyn Ruggieri, Lyn Finelli, Yoonyoung Choi

**Affiliations:** Department of Pediatrics, NewYork-Presbyterian Morgan Stanley Children’s Hospital, Columbia University Irving Medical Center, New York, New York, USA; Department of Pediatrics, Children’s Hospital of Philadelphia, Perelman School of Medicine at UPenn, Philadelphia, Pennsylvania, USA; Department of Pediatrics, Ann & Robert H. Lurie Children’s Hospital of Chicago, Chicago, Illinois, USA; Department of Pediatrics, Seattle Children’s Hospital, Seattle, Washington, USA; Department of Pediatrics, Johns Hopkins University School of Medicine, Baltimore, Maryland, USA; Department of Pediatrics, Children’s Hospital at Montefiore, Bronx, New York, New York, USA; Department of Pediatrics, NewYork-Presbyterian Morgan Stanley Children’s Hospital, Columbia University Irving Medical Center, New York, New York, USA; Mailman School of Public Health, Columbia University Irving Medical Center, New York, New York, USA; Department of Pediatrics, Children’s Hospital of Philadelphia, Perelman School of Medicine at UPenn, Philadelphia, Pennsylvania, USA; Department of Pediatrics, Ann & Robert H. Lurie Children’s Hospital of Chicago, Chicago, Illinois, USA; Department of Pediatrics, Seattle Children’s Hospital, Seattle, Washington, USA; Department of Pediatrics, Johns Hopkins University School of Medicine, Baltimore, Maryland, USA; Department of Pediatrics, Johns Hopkins University School of Medicine, Baltimore, Maryland, USA; Department of Pediatrics, NewYork-Presbyterian Morgan Stanley Children’s Hospital, Columbia University Irving Medical Center, New York, New York, USA; Department of Pediatrics, NewYork-Presbyterian Morgan Stanley Children’s Hospital, Columbia University Irving Medical Center, New York, New York, USA; Center for Observational and Real-World Evidence, Merck & Co., Inc., Rahway, New Jersey, USA; Center for Observational and Real-World Evidence, Merck & Co., Inc., Rahway, New Jersey, USA; Center for Observational and Real-World Evidence, Merck & Co., Inc., Rahway, New Jersey, USA

**Keywords:** escalation of respiratory support, healthcare-associated RSV, surveillance definitions, transmission index

## Abstract

**Background:**

Outbreaks of healthcare-associated respiratory syncytial virus (HA-RSV) infections in children are well described, but less is known about sporadic HA-RSV infections. We assessed the epidemiology and clinical outcomes associated with sporadic HA-RSV infections.

**Methods:**

We retrospectively identified hospitalized children ≤18 years old with HA-RSV infections in six children’s hospitals in the United States during the respiratory viral seasons October–April in 2016–2017, 2017–2018, and 2018–2019 and prospectively from October 2020 through November 2021. We evaluated outcomes temporally associated with HA-RSV infections including escalation of respiratory support, transfer to the pediatric intensive care unit (PICU), and in-hospital mortality. We assessed demographic characteristics and comorbid conditions associated with escalation of respiratory support.

**Results:**

We identified 122 children (median age 16.0 months [IQR 6, 60 months]) with HA-RSV. The median onset of HA-RSV infections was hospital day 14 (IQR 7, 34 days). Overall, 78 (63.9%) children had two or more comorbid conditions; cardiovascular, gastrointestinal, neurologic/neuromuscular, respiratory, and premature/ neonatal comorbidities were most common. Fifty-five (45.1%) children required escalation of respiratory support and 18 (14.8%) were transferred to the PICU. Five (4.1%) died during hospitalization. In the multivariable analysis, respiratory comorbidities (aOR: 3.36 [CI_95_ 1.41, 8.01]) were associated with increased odds of escalation of respiratory support.

**Conclusions:**

HA-RSV infections cause preventable morbidity and increase healthcare resource utilization. Further study of effective mitigation strategies for HA-respiratory viral infections should be prioritized; this priority is further supported by the impact of the COVID-19 pandemic on seasonal viral infections.

## Teaser

We identified 122 children with healthcare-associated (HA) respiratory syncytial virus infections; 55 (45%) required escalation of respiratory support and 18 (15%) were transferred to the Pediatric ICU. Further study of effective mitigation strategies for HA-respiratory viral infections should be prioritized.

## INTRODUCTION

Outbreaks of healthcare-associated respiratory syncytial virus (HA-RSV) infections are well described, particularly in neonatal intensive care units (ICUs), pediatric ICUs, and units caring for immunosuppressed children [1–4]. However, less is known about sporadic cases of HA-RSV infection and temporally associated outcomes. The objectives of this study were to describe the epidemiology of HA-RSV infections in children’s hospitals during multiple seasons, examine outcomes temporally associated with HA-RSV infections, and assess demographic characteristics and comorbid conditions associated with escalation of respiratory support. We also explored the applicability of the Centers for Disease Control and Prevention’s (CDC) National Healthcare Surveillance Network (NHSN) surveillance definitions for HA-upper respiratory tract infections (URTIs) and HA-pneumonia (PNA) in the children we identified with HA-RSV infections [5, 6].

## METHODS

### Study Design and Sites

A multi-center study was performed to identify HA-RSV infections in children 18 years of age and younger hospitalized in six academically affiliated children’s hospitals in the United States (median beds: 309 [interquartile range, IQR 197, 453]). HA-RSV infections were retrospectively identified during the respiratory viral seasons of October through April in 2016–2017, 2017–2018, and 2018–2019 and prospectively identified from October 2020 through November 2021. This latter surveillance period encompassed an RSV season asynchronous to the typical fall/winter season that was associated with the COVID-19 pandemic [7]. The RSV season of October 2019–April 2020 was excluded as sites diverted respiratory viral testing resources to testing for SARS-CoV-2 at the start of the pandemic. Each site obtained institutional review board approval or exemption to conduct this study with a waiver of informed consent.

### Study Definitions and Outcomes

#### HA-RSV Infections.

Defined as one or more new or worsening respiratory symptoms and a PCR test positive for RSV obtained ≥72 h after admission or if a child was readmitted, obtained ≤48 h after a previous, non-RSV-related hospital discharge. The following symptoms were collected on the day of the positive test for RSV (day 0): sore throat, cough, runny/stuffy nose, congestion, wheezing, headache, myalgia/body aches, malaise, fever >100.4°F (>38°C), shortness of breath, sputum production/increased sputum production, hoarseness, apnea/bradycardia (if in the NICU). Children were excluded if they had a positive RSV test within the past 6 months, if HA-RSV infection was diagnosed at an outside hospital prior to transfer to a study site, or if respiratory symptoms were present on admission, but respiratory viral testing was not performed within 72 h of admission.

#### Escalation of Respiratory Support.

Defined as one of the following: change from room air to supplemental oxygen (O_2_), increase in the fractional inspired (Fi)O_2_ on the same non-invasive respiratory support modality, change from non-invasive to invasive respiratory support, or increase in FiO_2_ and/or mean airway pressure (MAP) on the same invasive modality [8]. Non-invasive modalities included nasal cannula O_2_, high flow O_2_, continuous positive airway pressure (CPAP), or bi-level positive airway pressure (BiPAP). Invasive modalities included mechanical ventilation or extracorporeal membrane oxygenation (ECMO). Use of mechanical ventilation for ≤24 hours for a surgical procedure was not considered an escalation of respiratory support.

Other outcomes temporally associated with HA-RSV infections included the following: *transfer to the PICU* between day −2 and day +4; *initiation of contact, droplet, and/or airborne transmission precautions* on day 0, +1, +2, +3., or +4 to assess utilization of personal protective equipment (PPE) associated with HA-RSV infections; *delays in anticipated hospital discharge, administering chemotherapy, or scheduled surgical procedures*, identified by review of provider notes; and *overall hospital mortality* if death occurred prior to hospital discharge.

### Assessing Applicability of CDC NHSN URTI and PNA Surveillance Definitions

We explored if children with HA-RSV infections met existing CDC NHSN URTI and/or PNA surveillance definitions [5, 6]. To do so, signs and symptoms reported on day 0 and chest x-ray findings noted on radiology reports from day −2 to Day +2 were reviewed and compared with surveillance criteria (Supplementary Table 1).

### Data Collection From the Electronic Medical Record (EMR)

Children with HA-RSV infections were identified from the EMR and infection prevention and control surveillance (IP&C) logs. Site staff abstracted demographic and clinical characteristics including comorbid conditions [9], other viruses detected on day 0 and positive blood cultures obtained day −2 to day +2 from the EMR. To assess escalation of respiratory support from day −2 to day +4, respiratory support parameters were collected from day −10 to day +4. ICD-10 discharge codes were reviewed for the RSV-related codes J20.5, J21.0, J12.1, and/or B97.4. Death certificates were reviewed to determine if treating providers considered HA-RSV infections to have contributed to mortality.

### Data Analysis

To determine escalation of respiratory support, baseline respiratory support parameters from day −10 to day −3 were compared to respiratory support parameters used from two days before (day −2) the positive RSV test (day 0) to four days after (day +4) the positive RSV test. The interval day −2 to day +4 was considered the timeframe wherein an escalation of respiratory support would likely be due to HA-RSV infection [8, 10].

To assess the association of the community burden of RSV on HA-RSV infections, the median viral respiratory infection transmission index of each site (number of HA-RSV infections per 100 hospitalized community-acquired (CA) RSV infections) was calculated for each surveillance season [11, 12].

Demographic characteristics and selected comorbid conditions, including obesity [13], were described using frequencies, medians, proportions, and interquartile ranges (IQRs), when relevant. Unadjusted bivariate analyses were used to assess demographic and clinical factors associated with escalation of respiratory support. Variables were selected as candidates for the multivariable logistic regression model if they were associated with escalation of respiratory support in the unadjusted bivariate analysis (*P* < .05). We identified high collinearity between comorbid condition categories and therefore excluded the comorbidity category “other congenital or genetic defect” for children with high-risk comorbid conditions such as respiratory, cardiac, and/or neurologic conditions in the final multivariable model. A final multivariable logistic regression model was used to calculate adjusted odds ratios (aOR) and 95% confidence intervals (CI_95_) for factors associated with escalation of respiratory support (*P* < .05).

To assess the association between escalation of respiratory support and length of stay from RSV diagnosis to discharge, a Mann–Whitney *U* test was performed. Analyses were completed using Stata/BE 17.0 for Mac.

## RESULTS

### Study Population

During the study period, 122 children (median age 16 months [IQR 6, 60 months]) were identified with HA-RSV infections (median number of cases per site: 22 [IQR: 17, 25], range per site: 10–26). No HA-RSV clusters or outbreaks were identified by sites’ IP&C teams during the study period. The median onset of HA-RSV infections was hospital day 14 (IQR 7, 34 days). The demographic and clinical characteristics of children with HA-RSV infections are shown in Table 1. Overall, 78 (63.9%) children had two or more comorbid conditions of which cardiovascular, gastrointestinal, and neurologic/neuromuscular comorbidities were most common. Among 43 children 2 years of age or older with available weight and height, 4 (9.3%) were obese. Twenty-six children (21.3%) received respiratory support prior to the index hospitalization including mechanical ventilation (*n* = 6), non-invasive ventilation (*n* = 5), and supplemental oxygen (*n* =1 5). Thirty-six (29.5%) children had an RSV-related ICD-10 discharge code.

**Table 1.** Demographic Characteristics and Comorbid Conditions of Children with HA-RSV with and without Escalation of Respiratory Support

			Escalation of Respiratory Support		Unadjusted Odds Ratio (95% CI)^*^
		All, *N* = 122	Yes, (*n* = 55, 45%)	No, (*n* = 67, 55%)	
Demographic characteristics					
Sex, *n* (%)	Male	66 (54.1)	32 (58.2)	34 (50.8)	1.35 (0.66, 2.77)
Median age (IQR), months		16.0 (6, 60)	12.8 (4, 45)	22 (6, 96)	
Age, *n* (%)	≤12 months	53 (43.4)	27 (49.1)	26 (38.8)	**2.73 (1.03, 7.24)**
	13–60 months	40 (32.8)	20 (36.4)	20 (29.9)	2.63 (0.94, 7.3)
	>60 months	29 (23.8)	8 (14.6)	21 (31.3)	Ref
Race, *n* (%)	White	44 (36.1)	16 (29.1)	28 (41.8)	Ref
	Black/African American	26 (21.3)	15 (27.3)	11 (16.4)	2.39 (0.89, 6.43)
	Asian	4 (3.3)	2 (3.6)	2 (3.0)	1.75 (0.22, 13.65)
	American Indian, Alaska Native	1 (0.8)	1 (1.8)	0	NA
	Other	25 (20.5)^a^	12 (21.8)	13 (19.4)	1.62 (0.60, 4.38)
	Not answered/not specified/ Unknown	22 (18.0)	9 (16.4)	13 (19.4)	1.21 (0.43, 3.46)
Ethnicity, *n* (%)	Hispanic	33 (27.1)	12 (21.8)	21 (31.3)	Ref
	Non-Hispanic	81 (66.4)	38 (69.1)	43 (64.2)	1.55 (0.67, 3.56)
	Unknown	8 (6.6)	5 (9.1)	3 (4.5)	2.92 (0.59, 14.41)
Insurance, *n* (%)	Other, including Medicaid	69 (56.6)	30 (54.6)	39 (58.2)	Ref
	Private/parent employer/union	37 (30.3)	18 (32.7)	19 (28.4)	1.23 (0.55, 2.75)
	Uninsured/other/unknown	16 (13.1)	7 (12.7)	9 (13.4)	1.01 (0.34, 3.03)
Comorbid conditions, *n* (%)					
	Cardiovascular	42 (34.4)	23 (41.8)	19 (28.4)	1.82 (0.85, 3.86)
	Gastrointestinal	40 (32.8)	22 (40.0)	18 (26.9)	1.82 (0.85, 3.89)
	Neurologic/neuromuscular	39 (31.9)	23 (41.8)	16 (23.9)	**2.29 (1.05, 4.90)**
	Respiratory	36 (29.5)	24 (43.6)	12 (17.9)	**3.55 (1.56, 8.06)**
	Premature/neonatal	36 (29.5)	21 (38.2)	15 (22.4)	2.14 (0.97, 4.72)
	Other congenital or genetic defect^b^	35 (28.7)	24 (43.6)	11 (16.4)	**3.94 (1.71, 9.11)**
	Hematologic/immunologic/Rheumatologic	21 (17.2)	6 (10.9)	15 (22.4)	0.42 (0.15, 1.18)
	Malignancy	19 (15.6)	6 (10.9)	13 (19.4)	0.51 (0.18, 1.44)
	Renal/urologic	14 (11.5)	5 (9.1)	9 (13.4)	0.64 (0.20, 2.05)
	Metabolic	11 (9.0)	7 (12.7)	4 (6.0)	2.30 (0,64, 8.23)
	Miscellaneous^c^	3 (2.5)	1 (1.8)	2 (3.0)	0.60 (0.05, 6.82)
Number of comorbidities, *n* (%)					
	0	9 (7.4)	3 (5.5)	6 (9.0)	Ref
	1	35 (28.7)	8 (14.6)	27 (40.3)	0.60 (0.12, 2.92)
	2–3	47 (38.5)	25 (45.5)	22 (32.8)	2.27 (0.51, 10.18)
	≥ 4	31 (25.4)	19 (34.6)	12 (17.9)	3.17 (0.66, 15.12)

^*^Significant variables are bolded.

^a^Six were identified as Middle Eastern and two were identified as other combination not described.

^b^Other chromosomal and genetic defects included Down’s syndrome, congenital diaphragmatic hernia, and chromosomal abnormalities associated with known syndromes or unknown syndromes with congenital disorders described.

^c^Included chronic malnutrition, severe eczema, and hypotonia, cleft palate, and mandibular hypoplasia.

Seventeen (13.9%) children with HA-RSV infections had other respiratory viruses detected on day 0 including 11 with rhinovirus/enterovirus (RV/EV) and one each with influenza A H1 2009, influenza A H3, influenza B, parainfluenza 2, influenza A H3/coronavirus NL63, or influenza A H3/ RV/EV. Seven (11.5%) of 61 children for whom blood cultures were obtained had a positive blood culture including three coagulase negative staphylococci, two *Pseudomonas aeruginosa,* one methicillin-susceptible *Staphylococcus aureus*, and one *Enterococcus faecium.*

### Seasonality and Transmission Index of HA-RSV Infections

Sites identified 31, 33, 45, and 13 children with HA-RSV infection during the 2016–2017, 2017–2018, 2018–2019 surveillance seasons, and October 2020–November 2021, respectively. The median RSV transmission index for the 2016–2017, 2017–2018, 2018–2019 seasons, and October 2020–November 2021 was 0.79 [IQR 0.52, 1.98] (range per site 0.23–5.00), 0.66 [IQR 0.55, 1.48] (range 0.50–1.63), 1.18 [IQR 0.70, 2.48] (range 0.62–2.89), and 1.38 [IQR 0.67, 3.45] (range 0.53–8.33) HA-RSV cases per 100 CA-RSV cases, respectively.

### Outcomes Associated With HA-RSV Infections

Fifty-five (45.1%) children with HA-RSV infection required escalation of respiratory support, including 15 (12.3%) who transitioned from non-invasive to invasive support modalities (Table 2). From day −2 to day +4, 18 (14.8%) children were transferred to the PICU. Five (4.1%) children died during hospitalization with deaths occurring a median of 11 days (IQR 7, 47) after HA-RSV detection. One of the five death certificates identified RSV as a contributing factor.

**Table 2.** Clinical Outcomes Associated with HA-RSV Infections in Children

Outcome	HA-RSV, *N* = 122
Type of escalation of respiratory support, *n* (%)	
• None	67 (54.9)
• Change from room air to supplemental O_2_	19 (15.6)
• Increase in FiO_2_ on non-invasive modality	15 (12.3)
• Change from non-invasive to invasive support	15 (12.3)
• Increase in FiO_2_ on invasive modality	6 (4.9)
Transfer to pediatric ICU, *n* (%)	18 (14.8)
Delayed in anticipated hospital discharge, *n* (%)	14 (14.2)^a^
In-hospital mortality, *n* (%)	5 (4.1)

^a^Information regarding delayed hospital discharge was available for 106/122 (84.4%) children.

Bivariate analysis demonstrated that children who experienced escalations of respiratory support, as compared to those who did not, were more likely to be 12 months of age or younger and have respiratory, neurological/neuromuscular, and/or other congenital and genetic disorders (Table 1). In the multivariable analysis, respiratory comorbidities (aOR: 3.36 [CI_95_ 1.41, 8.01]) were significantly associated with increased odds of escalation of respiratory support when adjusting for age (as a continuous variable) and neurological/ neuromuscular comorbidities.

Ribavirin and intravenous immunoglobulin (IVIG) were prescribed for 6 (4.9%) and 5 (4.1%) children with HA-RSV infections, respectively. Those with escalation of respiratory support had significantly longer hospital stays after diagnosis of HA-RSV infection (median 32 days [IQR 12, 63]) than those without escalation (median 8 days [IQR 3, 26]), (*P* < .001). As documented in the EMR, 14 (13.2%) of 106 children with available data had evidence of a delay in anticipated hospital discharge. Further, while not relevant to all children, one child had delayed chemotherapy and two had delayed surgical procedures. On days 0, 1, 2, 3, or 4 contact, droplet, or both contact and droplet precautions were initiated for 14/106 (13.2%), 7/106 (6.6%), and 38/106 (35.8%) children, respectively. The remainder were on some type of isolation precautions when diagnosed with HA-RSV infections.

### Assessing CDC NHSN Surveillance Definitions for URTI and PNA

**Figure 1.**
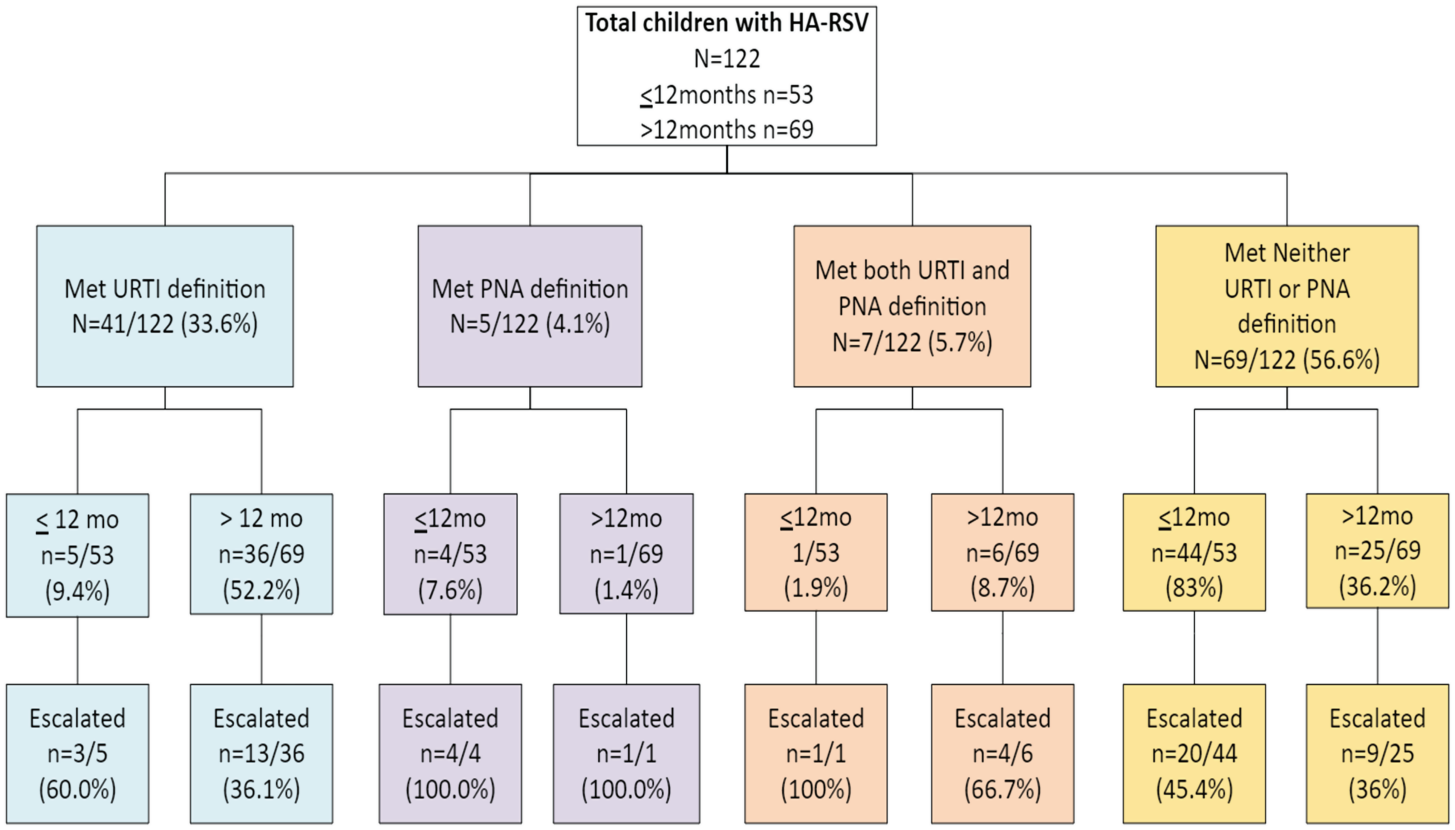
Association of HA-RSV CDC surveillance definitions for upper respiratory tract infection (URTI) and pneumonia (PNA) with escalation of respiratory support. The following are shown: number and percent of children 12 months of age and younger versus greater than 12 months who met the URTI definition, the PNA definition, both definitions, or neither definition and the number and percent of children who had their respiratory support escalated.

The most common symptoms exhibited by children with HA-RSV infections were fever, cough, tachypnea, and congestion, while apnea, erythema of the pharynx, purulent throat exudate, hoarseness, or change in sputum were noted for two or fewer children (Table 3). The number and proportion of children 12 months of age or younger vs. older than 12 months of age who met NHSN criteria for URTI and PNA are shown in Figure 1. Children with HA-RSV infections more frequently met the URTI definition (39.3%) than the PNA definition (9.8%); 56.6% of children did not meet either definition. Infants 12 months of age or younger were less likely to meet the URTI definition than older children (9.4%, 5/53 vs. 52.2%, 36/69, *P* < .001). Escalation of respiratory support occurred in a higher proportion of children who met the PNA definition (100%, 5/5) or met both the URTI and PNA definitions (83%, 5/6) compared with children who only met the URTI definition (39%, 16/41) or met neither definition (42%, 29/69), (*P* < .001).

**Table 3.** Frequency of Reported Criteria from CDC’s Surveillance Definitions for Healthcare-Associated Upper Respiratory Tract Infections and Pneumonia in Children ≤12 Months Versus >12 months With HA-RSV Infections (Criterion Included in Surveillance Definitions are Gray and Bolded)

	All HA-RSV, *N* = 122	≤12 months, *N* = 53	>12 months, *N* = 69
Upper respiratory tract infection criteria			
Vital signs			
Fever (>38°C)	57 (46.7%)	**20 (37.7%)**	**37 (53.6%)**
Tachypnea for age^a^	52 (42.6%)	24 (45.3%)	**28 (40.6%)**
Bradycardia < 100	4 (3.3%)	**2 (3.8%)**	2 (2.9%)
Hypothermia (<36°C)	3 (2.5%)	**3 (5.7%)**	0 (0.0%)
Apnea	1 (0.8%)	**1 (1.9%)**	0 (0.0%)
Respiratory symptoms			
Cough	55 (45.1%)	19 (35.8%)	**36 (52.2%)**
Congestion	42 (34.4%)	23 (43.4%)	19 (27.5%)
Nasal discharge/runny nose	24 (19.7%)	**8 (15.1%)**	**16 (23.2%)**
Sore throat	1 (0.8%)	0 (0.0%)	**1 (1.4%)**
Erythema of pharynx	0 (0.0%)	0 (0.0%)	**0 (0.0%)**
Purulent exudate in throat	0 (0.0%)	**0 (0.0%)**	**0 (0.0%)**
Hoarseness	0 (0.0%)	0 (0.0%)	**0 (0.0%)**
Pneumonia criteria			
Vital signs			
Apnea or tachypnea or retractions	58 (47.5%)	28 (52.8%)	30 (43.5%)
Fever (>38°C)^a^	57 (46.7%)	**20 (37.7%)**	**37 (53.6%)**
Tachypnea by age^b^	52 (42.6%)	**24 (45.3%)**	**28 (40.6%)**
Tachycardia >170	32 (26.2%)	**19 (35.8%)**	13 (18.8%)
Bradycardia <100	4 (3.3%)	**2 (3.8%)**	2 (2.9%)
Hypothermia (<36°C)	3 (2.5%)	3 (5.7%)	**0 (0.0%)**
Apnea	1 (0.8%)	**1 (1.9%)**	0 (0.0%)
Respiratory symptoms			
Cough	55 (45.1%)	**19 (35.8%)**	**36 (52.2%)**
Shortness of breath	31 (25.4%)	**14 (26.4%)**	**17 (24.6%)**
Physical findings			
Rales/ crackles or rhonchi/ coarse breath sounds	16 (13.1%)	9 (17.0%)	7 (10.1%)
Rales/ crackles or rhonchi/ coarse breath sounds or wheezing	19 (15.6%	10 (18.9%)	9 (13.0%)
Retractions	18 (14.8%)	**11 (20.8%)**	7 (10.1%)
Rales/crackles	9 (7.4%)	**5 (9.4%)**	**4 (5.8%)**
Rhonchi/coarse breath sounds^c^	9 (7.4%)	5 (9.4%)	4 (5.8%)
Wheezing	4 (3.3%)	2 (3.8%)	2 (2.9%)
White blood cell count			
Leukopenia ≤ 4000 WBC	26 (21.3%)	**3 (5.7%)**	**23 (33.3%)**
Leukocytosis ≥ 12 000 WBC	18 (14.8%)	**11 (20.8%)**	**7 (10.1%)**
Leukocytosis ≥ 15 000 WBC^a^	8 (6.6%)	**5 (9.4%)**	3 (4.3%)
Other			
New or increased sputum or increased suctioning requirements or change in character of sputum or purulent sputum	21 (17.2%)	13 (24.5%)	8 (11.6%)
Increased oxygen requirement^a^	25 (20.5%)	**14 (26.4%)**	**11 (15.9%)**
New or increased sputum	14 (11.5%)	**8 (15.1%)**	**6 (8.7%)**
Increased suctioning requirements	10 (8.2%)	**7 (13.2%)**	**3 (4.3%)**
Change in character of sputum or purulent sputum	1 (0.8%)	**0 (0.0%)**	**1 (1.4%)**

^a^The following criteria in the CDC surveillance definition were not collected: temperature instability in children ≤12 months, left shift for leukocytosis ≥ 15,000, nasal flaring with retractions or grunting, and increased ventilator demand.

^b^Age <2 months, respiratory rate (RR) ≥60; 2–12 months, RR ≥50; 13 months –5 years, RR ≥40; > 5 years, RR ≥ 30 [14].

^c^Rhonchi or coarse breath sounds were collected rather than the CDC NHSN criterion bronchial breath sounds.

## DISCUSSION

While HA-RSV infections have generally been among the most common HA-respiratory viral infections reported [15, 16], in this multicenter, 4-season study, we found that sporadic cases of HA-RSV were relatively rare. Nonetheless, these cases commonly required escalation of respiratory support and utilization of healthcare resources. We used EMR queries and IP&C logs to identify HA-RSV infections which enhanced case detection when compared to RSV-related ICD-10 discharge codes as only 30% of children with HA-RSV infections were identified by the latter. In addition, most cases of HA-RSV did not meet NHSN surveillance definitions for URTI and PNA. These findings highlight the need for better mechanisms to standardize and report healthcare-associated respiratory viral infections, potentially by improving NHSN surveillance definitions.

Over half of the children with HA-RSV infections were older than 12 months of age which is an older age range than previously described; this likely reflects the predominance of NICU clusters and outbreaks in previous reports [17, 18]. We found most children with HA-RSV infections had two or more comorbid conditions, most commonly cardiovascular, gastrointestinal, neurologic/neuromuscular, and respiratory comorbidities. Others have noted that children with HA-RSV infection were also more likely to be premature or immunocompromised [17–19]. Our study suggests that older, medically complex children with co-morbidities likely have longer hospitalizations and longer time at risk for HA-respiratory viral infections; they also may be more likely to be tested for HA-respiratory viruses compared with patients without co-morbidities. Further, there is a relative lack of preventive strategies, ie, palivizumab, for hospitalized infants or older children [20]. However, we did not assess the impact of RSV prophylaxis on HA-RSV infections which could be explored in future investigations. Mortality associated with HA-RSV infections was low as only one child who died had HA-RSV infection listed as contributing to death.

Fifty-five (45%) children with HA-RSV infections had an escalation of respiratory support including 15 (12.3%) children who required escalation from a non-invasive to an invasive modality. Others have also described a higher risk of mechanical ventilation in children with HA-RSV than CA-RSV infections [18] and reported that ~40%–62% of children with HA-RSV infections required respiratory support [18, 21]. However, others have similarly found that not all children with HA-RSV infections required escalation of support [18]. Such findings could inform families about the potential impact of HA-RSV infections in children.

Prevention of HA-respiratory viral infections is a quality improvement opportunity as these infections threaten patient safety and increase healthcare resource utilization. While there are not currently standardized measures to allow comparisons of HA-RSV infections over time or among hospitals, potentially the transmission index could be used for this purpose. For example, among nine children’s hospitals in Canada the transmission index for HA-RSV cases per 100 CA-RSV cases ranged from 2.8 to 13 from 1992 to 1994 [12].We speculate that differences in IP&C strategies, including variable use of contact precautions, were responsible for these observations. The transmission index did not decrease from October 2020 to November 2021 while universal mask use was in place for staff and visitors due to the pandemic. However, masks might not substantially impact RSV acquisition due to the role of contact transmission. Furthermore, testing practices for respiratory viral symptoms could have been different during the pandemic.

HA-RSV infections increase healthcare resource utilization. Investigators in Canada estimated that the costs of HA-RSV infections were 30% higher than the costs of CA-RSV infections [21]. Others found the median length of stay for HA-RSV infections was 10 days compared to 5 days for CA-RSV infections [19]. Similarly, we found increased healthcare resource utilization associated with HA-RSV infections including escalation of respiratory support, PICU admission, use of ribavirin and/or IVIG, delay in anticipated hospital discharge, and increased use of PPE.

A minority of children with HA-RSV infections met the NHSN surveillance definitions for URTI (39%) or PNA (10%). These surveillance definitions were developed for bacterial infections and respiratory viral infections may not cause the same severity of illness in some children. Additionally, some of the current criteria included in these definitions, eg, sore throat, erythema of the pharynx, purulent throat exudate, or hoarseness were absent or rarely reported in children with HA-RSV infections. Infants were less likely than older children to fulfill the surveillance definitions. We speculate this reflects the inclusion of more severe signs of illness in the URTI criteria for younger children (bradycardia, hypothermia, and apnea) and the exclusion of more common clinical manifestations of RSV (cough and congestion). Furthermore, two chest x-rays are required to diagnose PNA in children with underlying cardiac or respiratory comorbidities, but providers may be reluctant to obtain additional chest x-rays to limit children’s radiation exposure. Our findings suggest that revisions to the NHSN surveillance definitions for URTI and PNA should be considered to include symptoms consistent with HA-respiratory viral infections.

As this study demonstrates, HA-RSV infections continue to occur despite decades of recognition of the risk, complications, and cost of HA-respiratory viral infections and our understanding that enhanced surveillance, contact precautions, hand hygiene, environmental cleaning and disinfection, and staff education can reduce HA-RSV infections [17, 22–24]. While not specific for HA-RSV infections, exposure to an ill visitor is a potential modifiable risk factor [25], and visitor restrictions have been associated with reducing HA-respiratory viral infections [12]. In contrast, non-adherence with appropriate PPE, presenteeism of ill staff, and staff with asymptomatic infections increase the risk of HA-RSV infections [26]. Prevention strategies introduced during the COVID-19 pandemic including universal masking by staff and visitors, use of eye protection by staff (as the conjunctiva can be a route of acquisition), visitor restrictions, and increased attention to hand hygiene could also reduce HA-respiratory viral infections [27].

This study’s limitations include the generalizability of study findings as all sites were academically affiliated children’s hospitals. Our sample size was relatively small. The number of HA-RSV infections could have been overestimated as we defined HA-RSV infections as those that occurred ≥72 h after admission which could have included cases of CA-RSV infections with incubation periods >72 h [28] or could have been underestimated as we only assessed HA-RSV during the 2016–2019 respiratory viral seasons [15]. We did not assess the timing between symptoms onset and collection of the PCR test for RSV. PCR testing for HA-RSV infections and escalation of respiratory support are not standardized. Documentation of signs and symptoms in the EMR was likely incomplete. We did not assess the impact of ill caregivers, ill staff, or crowding on HA-RSV infections. The variability in sites’ transmission index could be due to the impact of these factors or due to different testing practices for CA-RSV. We did not assess initiation or escalation of antimicrobial therapy as measures of healthcare resource utilization. Outcomes, including mortality, temporally associated with HA-RSV infections may have been secondary to viral co-infections, bacteremia, or comorbid conditions. Signs and symptoms to assess NHSN surveillance definitions were only collected on day 0. Finally, we did not consider the applicability of these definitions in children with and without possible bacterial pneumonia.

In conclusion, we demonstrated that sporadic cases of HA-RSV infections were temporally associated with increased morbidity as some children required escalation of respiratory support and transfer to the PICU. Surveillance for HA-RSV infections could be improved by standardizing surveillance definitions for HA-respiratory viral infections. The transmission index could be used to trend HA-respiratory viruses and monitor the effectiveness of prevention efforts. Maternal vaccines for RSV and longer-acting monoclonal antibodies are in late-phase clinical development to prevent RSV in infants with high-risk conditions [29], including those potentially at risk for HA-RSV. More consistent surveillance and further study of effective mitigation strategies for HA-respiratory viruses should be prioritized; this priority is further supported by the impact of the COVID-19 pandemic on seasonal viral infections.

## Supplementary Material

piad030_suppl_Supplementary_Figure_S1

## Notes

**Financial support.** Funding for this research was provided by Merck Sharp & Dohme LLC, a subsidiary of Merck & Co., Inc., Rahway, NJ, USA. Part of study was presented at Spring SHEA, Colorado Springs, CO, in April 2022.

**Potential conflicts of interest.** L.S. serves on a Data Safety and Monitoring Board for a clinical trial sponsored by Merck & Co, Inc. All other authors: No reported conflicts.
