# Supplementary figures and images for: Healthcare-Associated Respiratory Syncytial Virus in Children’s Hospitals
Source: J Pediatric Infect Dis Soc. 2023 May 5;12(5):265–72. doi: 10.1093/jpids/piad030 (PMC10231385; doi:10.1093/jpids/piad030)

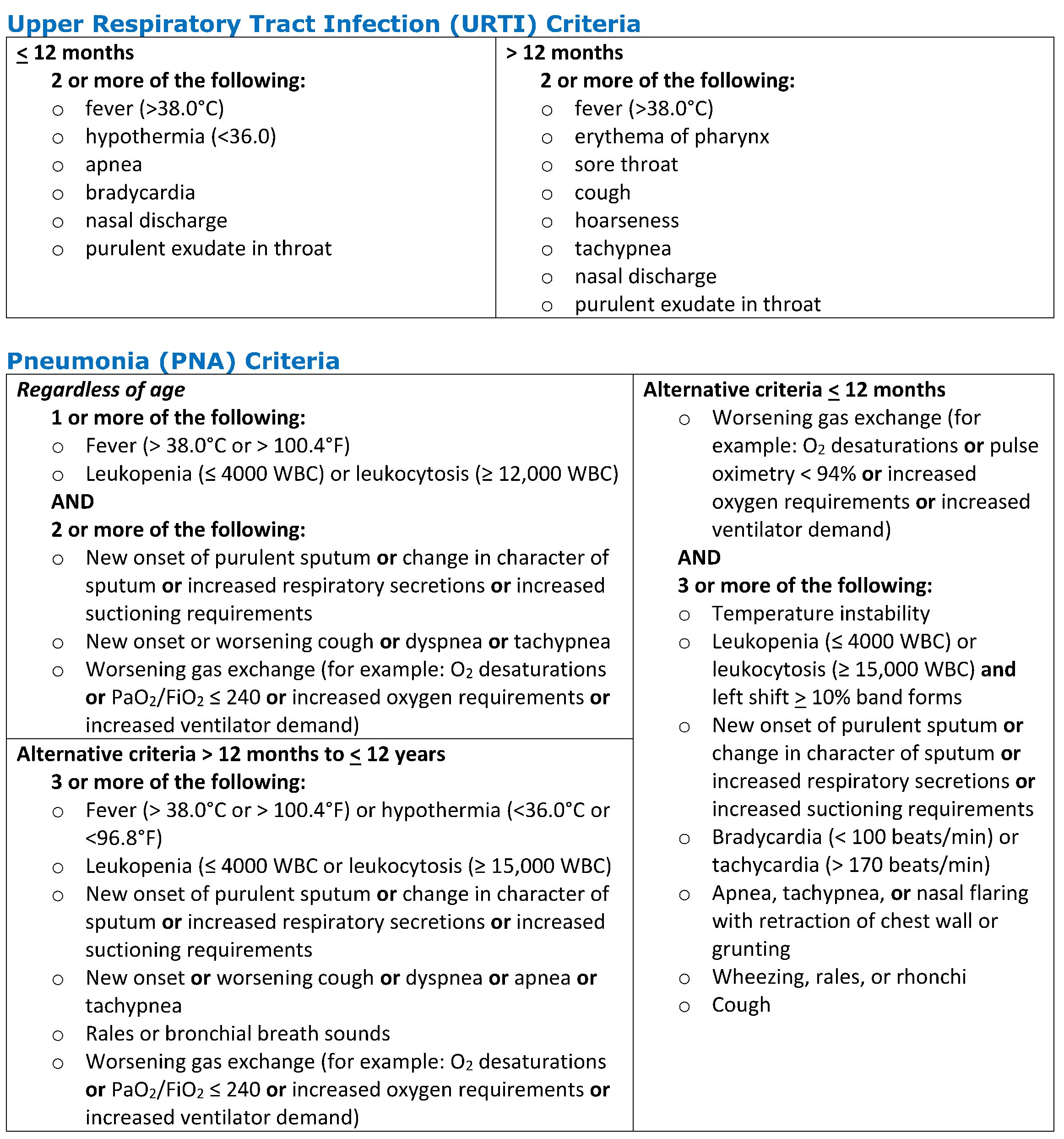

Supplement: piad030_suppl_Supplementary_Figure_S1 [file piad030_suppl_supplementary_figure_s1.jpeg]
